# Progress in Disease of Digestive Organs

**Published:** 1901-12-28

**Authors:** 


### PROGRESS IN DISEASE OF DIGESTIVE ORGANS.

(*Continued from page* 185.)

**Concealed Gastric Hæmorrhages.**—In the stomach we may either find copious bleeding or minute oozing from capillaries, ulcers, or growths. The quantity may be insufficient to alter the colour of the stomach contents, or we may get bright blood or coffee-ground material. Weber's guaiacum test will show blood which is invisible to the eye. Boas[12] notices that bleeding does not occur in the neuroses, nor in alterations of the secretions and benign dilatation. In ulcer and similar diseases it is sometimes present and sometimes absent, while in cancer he found blood in every case. Hale White[13] brings forward an interesting theory that many cases of supposed gastric ulcer with hæmatemesis in young women are not ulcer at all, but an oozing from superficial erosions, or possibly analogous to vicarious menstruation. Even when the hæmorrhage is fatal there is sometimes no ulcer to be seen *post-mortem*, and surgeons record many cases of operation where no ulcer was found. It is noteworthy that so-called gastric ulcer though much more common in women than in men is far more fatal in men and that many of these chlorotic bleeders go on to the menopause without perforation, or the mechanical effects of ulcer such as adhesions or stenosis. However, at present the diagnosis between bleeding from an ulcer and bleeding without one is very difficult. Hæmatemesis occasionally occurs also in acute attacks of stenosis of the intestines. Atixier and Viannay[14] mention a case due to adhesions and obstruction of the bowel near the ilio cæcal valve which died in spite of operation. The bleeding was due to the congestion of the mucous membrane from the constriction and toxic changes. Such hæmorrhage is always a most unfavourable symptom.

**Gastric Tetany** or tetany occurring in dilatation of the stomach is very fatal, and its causes very little understood. Halliburton and McKendrick[15] report the results following the injection into animals of an extract of the stomach contents. The patient suffered from dilatation due to stenosis after an ulcer, and had frequently washed out his stomach. After a feeling of nausea, headache, tingling and numbness down the arms and fingers he fell into a comatose state for 36 hours. A strong clonic contraction of the arms and hands was present and the urine contained albumen, acetone, and later on sugar. By purgatives and high rectal injections he was relieved and finally cured by pylorectomy. The writers found that a toxic substance was present which caused a marked fall of blood pressure and slowing of the heart beat when injected into animals. It had a marked acid reaction and was soluble in alcohol and salt solution. Others have ascribed the attacks to dehydration from the vomiting, and others to a peptotoxin, or to a poison produced by the excess of hydrochloric acid supposed to be present in these patients. Cassaet[16] has obtained two extractives, one of which produced convulsions, and the other caused coma. He holds that the poisons are derived from albuminous food substances which have been partly changed into peptones, and absorbed in that state. The treatment of fermentative conditions should, according to J. C. Hemmeter,[17] be directed to restore the motor power. Lavage, however, is of great value, and intragastric faradism helps greatly to restore the muscular activity. Carbohydrate food must be left off for a time, and a finely divided proteid diet substituted with, if necessary, the administration of hydrochloric acid. Carl von Noorden washes out the stomach every night, two hours after the last meal, after which a large dose of chloroform water is given. Thymol is taken after each meal, and at the beginning of treatment a second lavage is employed in the morning for a few days.

12 Med. Rev., July 6. 13 Lancet, June 29. 14 Amer. Jour. Med. Sc., April. 15 Brit. Med. Jour., June 29. 16 Brit. Med. Jour., May 11. 17 Amer. Jour. Med. Sc., August.

